# A quantitative comparison of micro-CT preparations in Dipteran flies

**DOI:** 10.1038/srep39380

**Published:** 2016-12-21

**Authors:** Peter Swart, Martina Wicklein, Dan Sykes, Farah Ahmed, Holger G. Krapp

**Affiliations:** 1 Department of Bioengineering, Imperial College London, UK; 2 Department of Neuroscience, Physiology and Pharmacology, University College London, UK; 3 Imaging and Analysis Centre, Natural History Museum, Cromwell Road, London, UK; 4 Henry Moseley X-ray Imaging Facility, University of Manchester, Manchester, UK.

## Abstract

X-ray-based 3D-imaging techniques have gained fundamental significance in research areas ranging from taxonomy to bioengineering. There is demand for the characterisation of species-specific morphological adaptations, micro-CT (μCT) being the method of choice in small-scale animals. This has driven the development of suitable staining techniques to improve absorption-based tissue contrast. A quantitative account on the limits of current staining protocols for preparing μCT specimen, however, is still missing. Here we present a study that quantifies results obtained by combining a variety of different contrast agents and fixative treatments that provides general guidance for μCT applications, particularly suitable for insect species. Using a blowfly model system (*Calliphora*), we enhanced effective spatial resolution and, in particular, optimised tissue contrast enabling semi-automated segmentation of soft and hard tissue from μCT data. We introduce a novel probabilistic measure of the contrast between tissues: PTC. Our results show that a strong iodine solution provides the greatest overall increase in tissue contrast, however phosphotungstic acid offers better inter-tissue discriminability. We further show that using paraformaldehyde as a fixative as opposed to ethanol, slows down the uptake of a staining solution by approximately a factor of two.

Detailed knowledge of the shape and arrangement of anatomical structures are crucial in research areas ranging from taxonomy and systematics to biomechanics and physiology. Ideally such data should be gathered in structurally intact animals, thereby minimising loss or deterioration of morphological features. For comparative studies, this would help to identify and describe homolog structures as well as their species-specific morphological adaptations. Conventional techniques used for morphological studies including light or confocal microscopy provide the required spatial resolution and tissue contrast, but the preparation of specimens is exceedingly time consuming. It may also lead to partial destruction of the specimens, resulting in distortions or loss of morphological information. The excessive amount of time spent in preparing microscopy datasets quite often prohibits studying a sufficiently large number of specimens required for a thorough statistical analysis. To overcome these limitations, novel techniques are needed that provide sufficient resolution without loss of information or distortion, enable high throughput by means of computer-aided analysis, and preserve the specimens for collection or future comparative studies. Such techniques would substantially benefit research in all areas that rely on functional anatomical data.

X-ray micro-computed tomography (micro-CT) in conjunction with differential contrast enhancement has been shown to address these demands^1^,^2^,^3^,^4^. This technique resolves small structural details down to a scale of a few micrometres while enabling high throughput and the possibility for automated image analysis of data obtained from structurally intact specimens. Besides dehydration and fixation, neither a major dissection nor sectioning of the specimens are required, which minimises the distortions and thus the loss of structural information. The only drawback of the technique is that transmission X-ray imaging provides little native soft-tissue contrast. Image formation, in most cases, is simply based on the absorption of X-rays as they pass through the tissue. The factor determining contrast is the difference in the linear X-ray attenuation coefficient between tissues. This coefficient is large for metal and bone, and small for water and soft tissues.

Recent improvements in micro-CT (μCT) imaging quality were made by using different fixatives as well as different contrast agents^2^,^3^,^4^,^5^,^6^,^7^,^8^,^9^,^10^,^11^,^12^,^13^,^14^. Iodine and phosphotungstic acid (PTA) have proven to be potent contrasting agents and to provide versatile tissue stains for soft tissue in both vertebrate and invertebrate specimens^2^. While osmium-tetroxide has been shown to be an effective staining agent^10^, the extra difficulty in handling the substance due to its toxicity was a key prohibiting factor. Quantitative studies revealing the contrast gains with different staining regimes are still lacking. To provide this necessary quantitative analysis on μCT preparations, we introduce a novel method of quantifying tissue contrast.

In this account we focused on a comparison between the efficacy of different staining regimes, including PTA and iodine contrast agents (stains), in fixed and ethanol-dehydrated specimens. We compared the staining progression through the specimen as well as the uptake of the stain into different tissue types. This information will greatly enhance the ability to customise staining times and protocols for a broad range of applications. In our analysis we used the blowfly *Calliphora vicina* as a model system and concentrated on a subset of morphologically diverse tissues, including cuticle, nervous tissue and muscles. The contrast our staining method achieved was generally high enough to enable automated segmentation of the image data. Automatic segmentation greatly enhances the efficiency of the anatomical analysis in the specimens studied. We quantified the contrast achieved by different regimes in a variety of tissue types using traditional contrast measures as well as our own novel probabilistic contrast measure. Further, we were able to design customised staining procedures targeting specific structures or tissue types.

## Materials and Methods

### Animals

Five adult *Calliphora vicina* were collected from our laboratory colony. The colony was maintained at approximately 23 °C with a light:dark cycle of 12:12 h. Adults were provided with sugar and water; larvae were fed on pork liver. For the experiments, we selected both male and female flies between 2 and 4 days old.

### Preparation of animals

Adult flies were cold anesthetised and the legs, wings and abdomen were removed. To facilitate penetration of the tissue by the fixative and the staining solution we also removed the distal third of the thorax, which had no discernible effect on the structural integrity of the proximal thorax or head. Dissections were performed with the specimen entirely submerged in 100% ethanol to prevent forming of air bubbles within the specimens. Immediately following the dissection, the flies were transferred to vials of 100% ethanol where they were left for 2 days to fix at room temperature. The same procedure was performed on flies fixed in a 2% paraformaldehyde (PFA) solution, except these were left for 1 day at room temperature. The difference between fixation times for ethanol and PFA was based on preliminary tests suggesting that 1 day in ethanol was insufficient to adequately fix the tissue.

### Staining of specimen for scanning

Two different fixatives (a) were applied alongside two separate staining solutions (b) to test four different protocols:

- We compare a 100% ethanol fixative with a paraformaldehyde (PFA) fix; 2% PFA in Sorensen’s buffer (0.2 M NaH_2_PO_4_*2H_2_0 (27.6 g/l) + 0.2 M Na_2_HPO_4_*2H_2_0 (35.6 g/l)).
- 0.15% Iodine solution in 100% ethanol compared with 0.5% PTA in 70% ethanol^2^.

In addition to the two iodine-stained specimen, we added one fly fixed in ethanol (as above), stained in 1% Lugol’s solution (1% iodine and 2% potassium iodide mixed in distilled water). Table 1 outlines the combination of staining and fixing methods applied to the flies.

**Figure 1:**
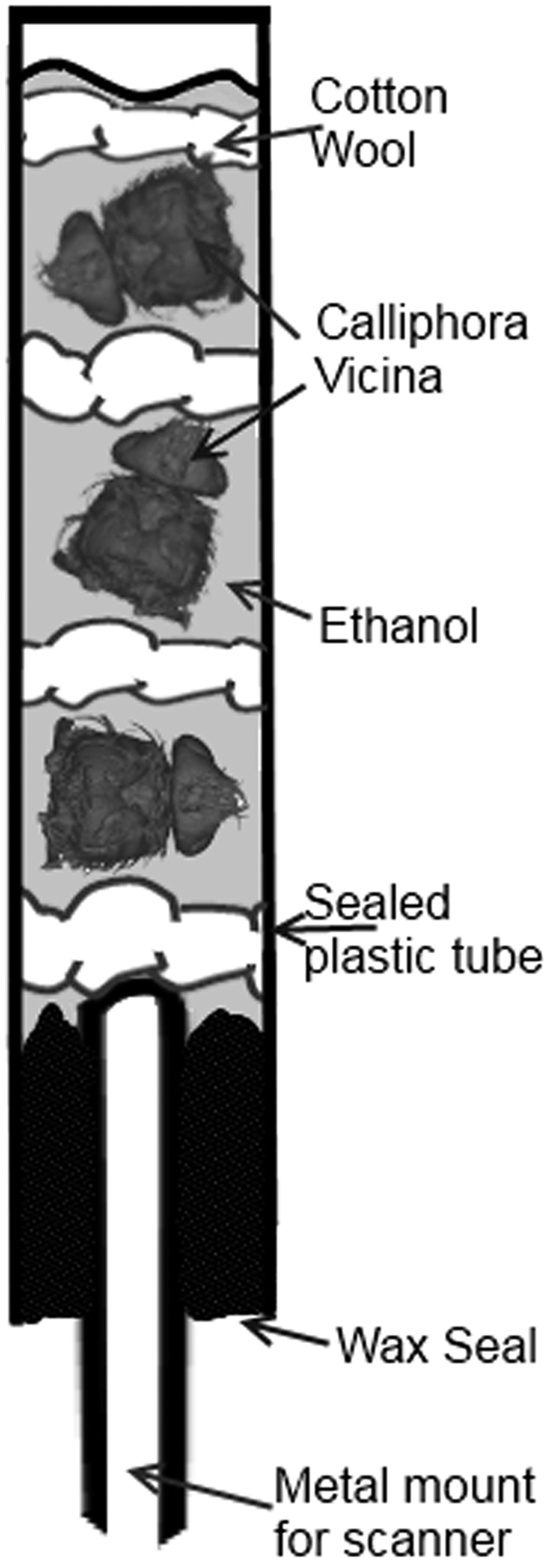
Schematic of scanning setup. Further explanation in text.

Specimens were stored at room temperature (23 °C) in the solutions specified above for 3 days. 30 minutes prior to CT scanning, samples were washed three times, (once every 10 minutes) in 70% ethanol. Three specimens (of the same stain) were scanned simultaneously in small plastic tubes filled with 70% ethanol, separated by cotton wool (Fig. 1). The cotton wool was inserted between the flies to simplify a software-based separation of individual specimens once the entire 3D volumes of the tubes containing three flies had been reconstructed. Each batch of three specimens was scanned every day at the same time for 5 days – this reflects 3, 4, 5, 6, and 7 days of staining duration. After scanning, each specimen was returned to its 5 ml vial containing a refreshed staining solution and left at room temperature in order to continue uptake of the staining solution. The precise staining solutions are described in the following, and summed up in Table 1 below: 0.5% PTA solution is made by dissolving 500 mg of PTA in 106 ml of 70% ethanol. 2% PFA solution is made by dissolving 2 g of PFA in 95 ml of Sorensen’s buffer (note: dissolved by heating to approximately 50 °C and stirring overnight with a magnetic stirrer). 0.15% iodine solution is made up by dissolving 1 ml of 0.5 M aqueous iodine in 100 ml of 100% ethanol (total: 0.15% iodine, 1.2% water, 98.65% ethanol). 1% Lugol’s solution consists of 1% elemental iodine (I2) and 2% potassium iodide (KI) in distilled water.

### Μicro-CT scanning and settings

CT scans were performed using a Nikon Metrology HMX ST 225. All samples were scanned using the same X-ray energy spectrum (Bremsstrahlung created by 120 keV electrons on a molybdenum target, the electron current was 180 μA). Each scan lasted 26 minutes, acquiring 3,142 projections with an exposure of 500 ms per projection.

### Analysis and reconstruction of scans

The raw scan datasets were imported into CT-Pro 2.1 (Nikon Metrology, Tring, UK) and set to reconstruct after the centre of rotation was determined automatically. The scans were reconstructed using a filtered back projection algorithm. Across the total of 10 scans, reconstructed voxel sizes varied between 8.4 μm^3^ and 13.6 μm^3^. This was largely dependent upon how close the flies could be arranged to each other within the plastic tube, which determined the field of view of the scanner. The resulting reconstructions were exported in a DICOM format and imported into Mimics (v14.0, Materialise NV, Leuven, Belgium). Using Mimics, the data was segmented into the different tissue types described below, and rendered in 3D. MatLab (v7.14, MathWorks Inc., Natick, MA) was used for calculation of contrast values.

### Simple contrast ratio

The 3D volumes were reconstructed as 16-bit greyscale values, used to determine the contrast ratio, which was quantified as the difference in mean greyvalue between patches of background and tissue, divided by the mean background greyvalue:


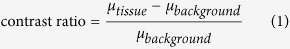


We assumed that the background was a homogenous liquid and therefore all variations in background signal depended solely on the scanning or reconstruction processes. Greyscale values used in calculating contrast ratios for tissues were based on the average across all individual measurements.

**Figure 2:**
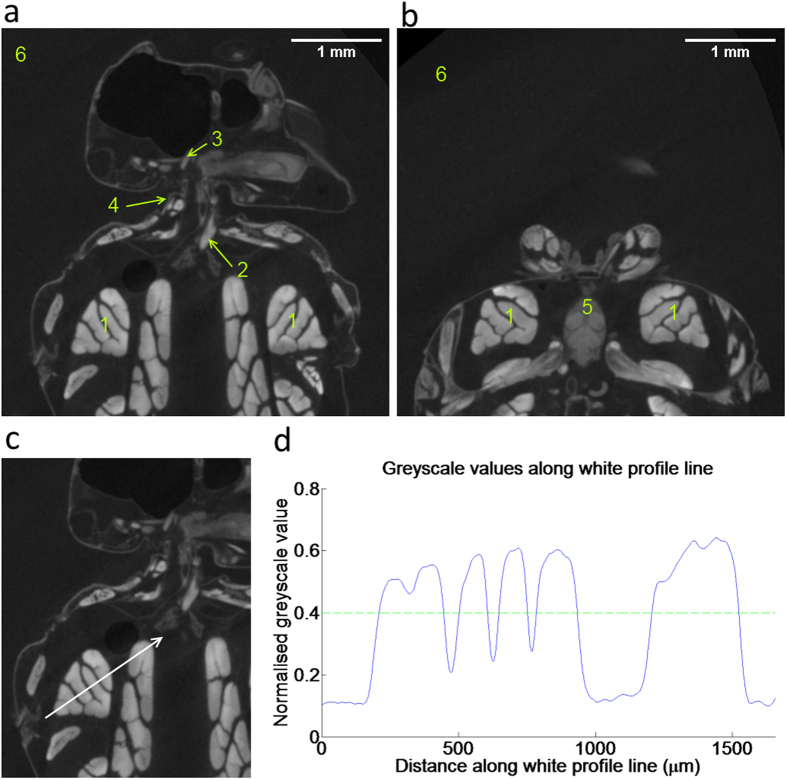
Soft tissue measurement locations. (**a**) Horizontal section of the upper thorax and head; labelled are the tissue types used for the measurements: The large muscle (1) greyvalue measurements are taken from the dorsoventral flight muscles. The small muscle (2) greyvalue measurements are taken from the oblique horizontal neck muscles. The thick cuticle (3) greyvalue measurements are taken from the pronotal apodeme. The thin cuticle (4) greyvalue measurements are taken from the cervical sclerite. The background (6) greyvalue measurements are taken from the ethanol filled spaces surrounding the fly. (**b**) Horizontal section of the upper thorax, anterior to the slice shown in (**a**). A subset of the 7 neuropils included in the characterization of neural tissue (5) is visible in this slice. Additionally, the background values (6) and the large flight muscles (1) are visible in this slice. (**c**) Horizontal slice with an arrow superimposed on the large flight muscles. The arrow forms the x-axis of the graph shown in; (**d**) Greyscale profile along the white line in (**c**). The y-axis gives the corresponding normalised greyscale values.

For each tissue type, between 6 and 8 contrast measurements were taken and averaged to give the mean tissue greyvalue, 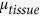. A large area of pixels in the background region in Fig. 2a (region 6) was averaged, yielding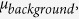 which is the greyvalue for the ethanol solution surrounding the fixed tissue. These were calculated for each stain/fix combination, and for each day of scanning. This contrast ratio is a simple measure and easy to obtain, and therefore is used to determine the stain progression as a function of time.

### Probabilistic tissue contrast

To give a more detailed assessment of the threshold-based discriminability between tissues, we introduced a new contrast measure outlined below. This measure is grounded in statistical analysis and requires a larger sample size for reliable calculations, which will be addressed at the end of this section.

We assume that Gaussian distributions:


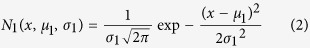



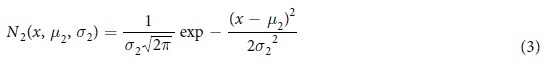


Represent the distributions of the greyvalues of two tissues, where *σ*_2_ < *σ*_1_. We define the probabilistic tissue contrast (PTC) as follows:


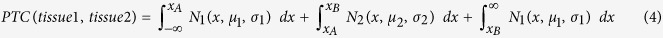


where *x*_*A*_ < *x*_*B*_. *x*_*A*_ and *x*_*B*_ represent the two intersection points of probability distributions *N*_1_ and *N*_2_. These intersection points can be found by equating formulae (2) and (3):


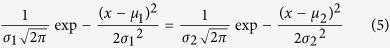


Solving for *x*, yields:


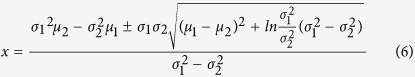


These are two real-valued intersection points, except in the trivial case *σ*_2_ = *σ*_1_, where there exists only one intersection point at 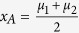. In this case equation (4) simplifies to:


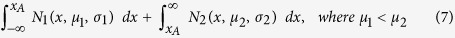


**Figure 3:**
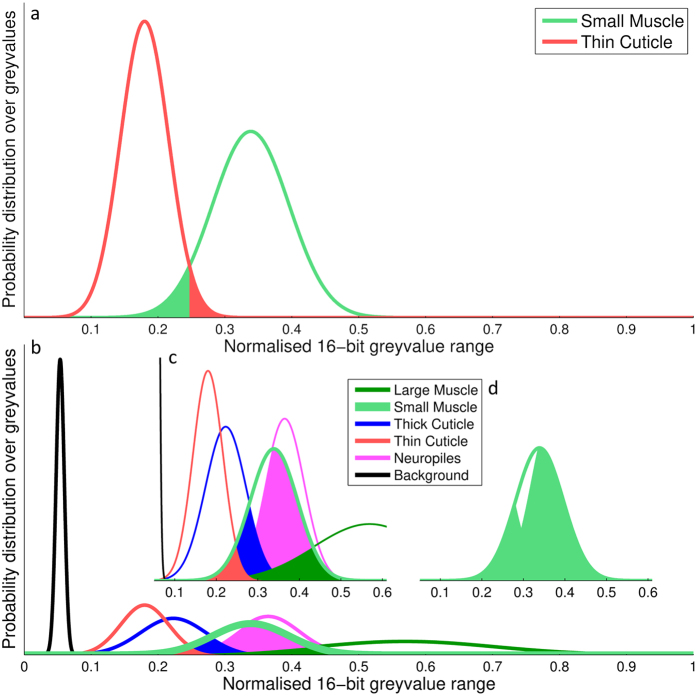
Probabilistic contrast measure example. (**a**) Panel a shows a simplistic two tissue discrimination. On the left the probability distribution for the thin cuticle tissue over a 16 bit greyvalue range is shown. The probability distribution on the right shows the greyvalues of small muscle tissue type. The highlighted section indicates the overlap between the two distributions and therefore the discrimination error when applying a blanket threshold – in this example the threshold would be at around 0.25 on the x-axis. The area of the highlighted region gives the probabilistic tissue contrast (PTC) as a value between 0 and 1. (**b**) Panel b shows the same comparison of tissue probability distributions with all other measured tissue types included. The small muscle tissue type is contrasted against all other tissue types to determine its overall discriminability. (**c**) Panel c shows the same distribution as in panel b, zoomed-in into the region of interest which shows the pairwise overlap between the probability distributions of the small muscle tissue type and all other tissue types. (**d**) Panel d shows the cumulative overlap between the small muscle tissue type probability distribution and all others, giving the overall PTC for the small muscle tissue type. In this case, the value is close to 1, giving a low degree of discriminability.

The PTC reflects the probability that, given a sample taken randomly from the union of both distributions, the sample is wrongly classified; i.e. it was assigned to the distribution other than the one it actually belongs to. In this way, a low PTC value (close to zero) represents a high degree of discriminability between the two tissues. This is demonstrated in the examples shown in Fig. 3.

This contrast measure gives a detailed and accurate view of the discriminability between tissues, however it also requires a large sample size. It will therefore only be used on the fully stained (day 7) scanning conditions to compare the efficacy of different staining regimes in detail.

**Figure 4:**
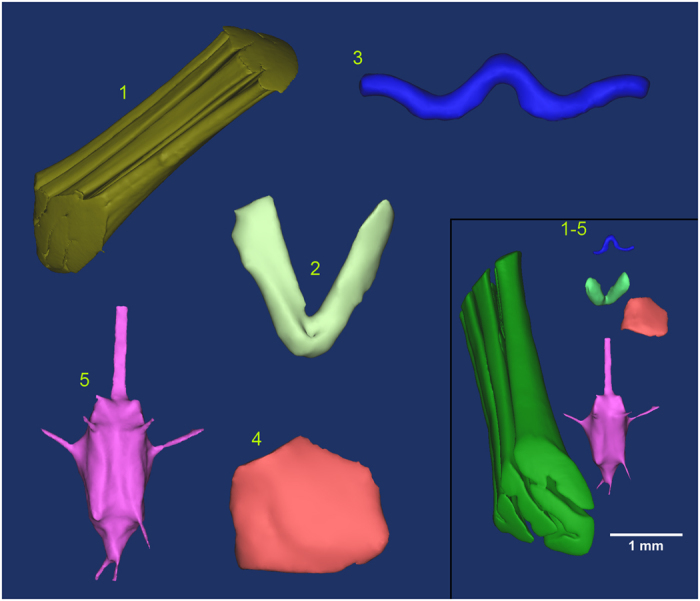
3D semi-automatic segmentation examples. (**1**) The right dorsoventral flight muscle (large muscle tissue type), segmented by thresholding out the lower background values, and rendered in 3D using Mimics (v14.0, Materialise NV, Leuven, Belgium). (**2**) A pair of small neck muscles, the oblique horizontal pair, as described by Strausfeld, 1987. The resulting volume measurements of these are halved to get the volume of a single muscle. (**3**) A portion of thick cuticle, the pronotal apodeme. Located inside the head, it serves as an attachment point for a number of neck muscles/tendons. (**4**) A sheath of thin cuticle which forms a covering over a group of neck muscles. (**5**) Seven neuropils with surrounding neural tissue and axons extending from the region. (**1**–**5**) Shows all 5 tissues with their relative sizes and orientations. The scale bar applies only to this sub-image.

Given sufficient staining, the segmentation process used to create the renderings shown in Fig. 4 is largely automatic. Manual input is only required when choosing a greyscale threshold and defining a region of interest, shown in Fig. 2c and d, where a normalised threshold value of approximately 0.4 is chosen. Choosing a threshold becomes trivial due to the large difference in contrast between the tissue types and the background; this enables the segmentation of a clearly defined single muscle, that is easily distinguished from the background tissues (see Fig. 4, image 1).

### Tissue types

For comparison, we selected specific anatomical structures and provide a size parameter in addition to the contrast values characterising each of the following tissues types:

1. For large muscles, the right dorsoventral flight muscle was selected (Figs 2 and 4), and the volume of the muscle measured.
2. For small muscles, a pair of neck muscles, the oblique horizontal muscles [9] (Nomenclature relating to neck muscles and associated cuticula is taken from Strausfeld, 1987) were selected (Figs 2a and 4) and their volume measured. The volumes of both muscles combined was measured and subsequently halved, because the close proximity of the muscles made it difficult to separate them individually.
3. For thick cuticle, the pronotal apodeme^15^ was selected (Figs 2a and 4). It is approximately cylindrical in shape, therefore we measured its diameter.
4. For thin cuticle, the cervical sclerite^15^ was selected (Figs 2a and 4). It forms a covering sheet over a group of muscles, therefore we measured sheet thickness.
5. For neural tissue, we analysed various neuropils within the thoracic ganglion (Figs 2b and 4). These are 7 neuropils, interconnected and branching out to various locations in the head, thorax and abdomen. The total volume of these neuropils was measured.

Care was taken to select candidate structures for each tissue type that are in close proximity to each other. To compare the uptake of the contrast agent across the five scanning days, we minimised the distance between the five different tissues. Therefore, we have chosen flight muscles closest to the head, to be compared with neck muscles and cuticular structures in the neck region. Lastly, to assess neural tissue, we have selected neuropils in the prothoracic ganglion in close proximity to the selected flight and neck muscles. Choosing neighbouring muscles also helped to reduce any variability from CT reconstruction due to varying distance from the centre of rotation.

### Stain Uptake

In addition to measuring greyscale values, we estimated the stain uptake by diffusion through the background ethanol. We approximated the uptake rate close to the incision by a sigmoidal or error function with respect to time, according to a one-dimensional solution to Fick’s law^16^; modelling this, however, is beyond the scope of this study. The one-dimensional simplification assumes the axis used for the uptake is measured from the incision along the length of the specimen toward the head. The stain uptake is measured in two different tissues: eyes and flight muscles.

We define the stain uptake rate as the distance the stain travels by diffusing through the fluid inside the fly per day. This was measured at the eyes; in terms of distance from the incision, this is approximately 5 mm, but varies from fly to fly. By selecting the same 2D plane in each 3D stack, the total stained circumference of the eye was measured for each day. In this measurement we assume that the ommatidia in the eyes are stained immediately once in contact with the stain. It is assumed that stain uptake through the spiracles, i.e. the entrance to the fly respiratory system, is negligible compared with uptake through the incision.

We measure the stain uptake rate in the large flight muscles by measuring the stained volume as a function of the number of days stained. This was done for the specimens treated with ETH-PTA and PFA-PTA to assess the effect of the paraformaldehyde fixative on stain uptake rate.

## Results

### Tissue segmentation

In this section, we provide a quantitative estimate of the efficacy of each staining procedure. Contrast values were calculated for the five tissue types specified previously. The following direct measurements give the size parameters for each tissue type:

1. The volume of the flight muscle (Fig. 4): μ = 0.99 mm^3^, s.d. = 0.19 mm^3^, N = 5
2. The volume of the neck muscle (Fig. 4): μ = 0.0043 mm^3^, s.d. = 0.0011 mm^3^, N = 5.
3. The diameter of the approximately cylindrical pronotal apodeme or “thick cuticle” (Fig. 4): μ = 57 μm, s.d. = 6.7 μm, N = 5.
4. The sheet thickness of the cervical sclerite, or “thin cuticle” (Fig. 4): μ = 25 μm, s.d. = 3.0 μm, N = 5.
5. The total volume of the 7 neuropils in the thoracic ganglia (Fig. 4): μ = 0.056 mm^3^, s.d. = 0.011 mm^3^, N = 5).

For each tissue type, the entire tissue was segmented (see Fig. 4) using a semi-automatic method. This entails automatically thresholding out each tissue type within a manually specified region. The mean and standard deviations were calculated from between 10^3^ and 10^7^ measurements, depending on tissue type: For large flight muscles, N~10^7^; small neck muscles, N~10^4^; thick cuticle, N~10^3^; thin cuticle, N~10^3^; thoracic ganglion neuropils, N~10^5^.

### Probabilistic tissue contrast measurements

The probabilistic tissue contrast (PTC) for each tissue was calculated.

**Figure 5:**
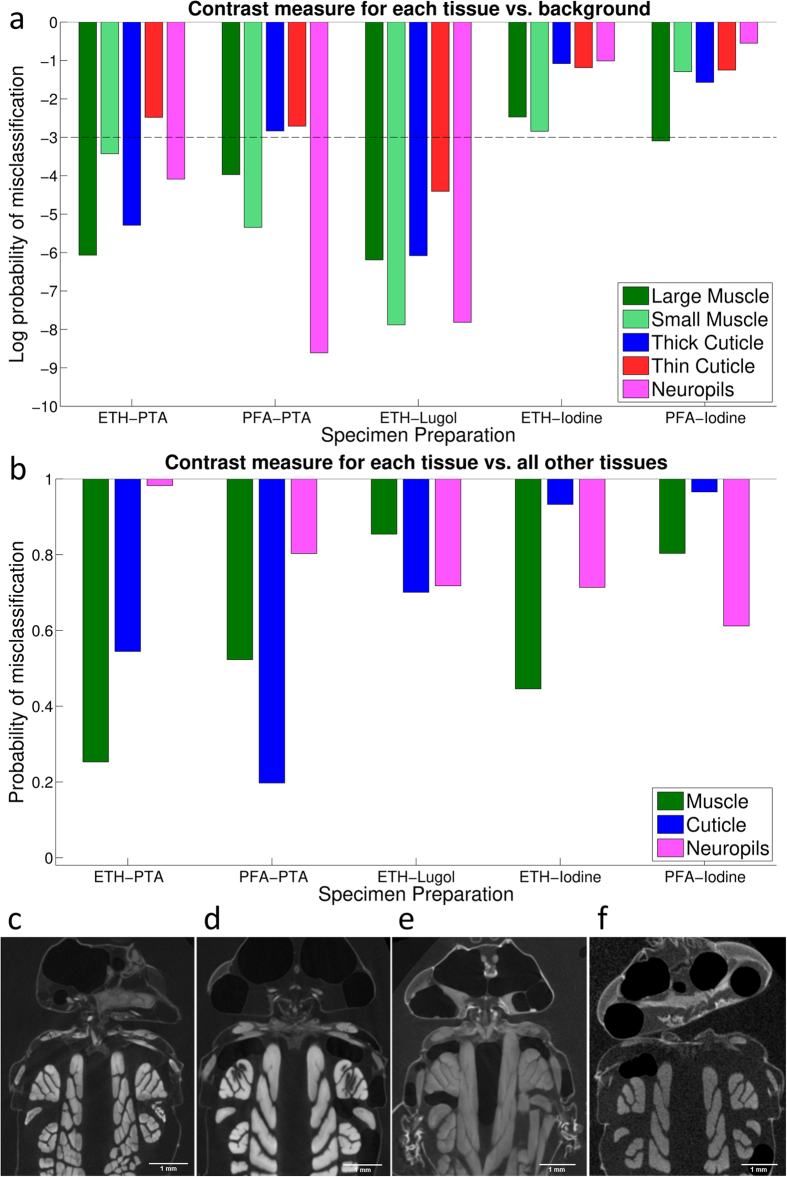
A quantitative and qualitative comparison of the 5 preparation methods after 5 days of staining. (**a**) A comparison of the day 7 probabilistic tissue contrast (PTC) and the background across the 5 preparation methods for each of the five tissue types. Labels along the x-axis show the abbreviations for the staining regime. The y-axis shows the log likelihood of misclassification for each tissue type i.e. its PTC. As a rough guide, 10^−3^ is the level at which automatic thresholding becomes possible, as indicated with a dotted line. Subfigures (**a**) and (**b**) have been arranged such that the larger the bar, the better the contrast for the respective tissue type. (**b**) A comparison of the day 7 probabilistic tissue contrast for each tissue and all other tissue types. For simplicity, the two muscle types and the two cuticle types have been combined. (**c**–**f**) show horizontal slices of ETH-PTA (**c**), PFA-PTA (**d**), ETH-Lugol (**e**), PFA-Iodine (**f**) at approximately the same depth and orientation. The slice for ETH-Iodine exhibits similarly poor contrast to that of PFA-Iodine and is omitted for brevity.

Low concentration (0.15%) iodine solution shows generally poor contrast as shown in Fig. 5. A PTC of 10^−3^ is the approximate level at which automatic thresholding becomes possible (indicated in Fig. 5a). This means that 1 in every 1000 voxels will be misclassified when segmenting out a particular tissue. For the low concentration iodine solution, only the muscles come close to meeting this criterion. Higher concentration (1%) Lugol’s solution exhibits very good contrast for all tissues against the background greyvalues, allowing for automatic segmentation. Both PTA solutions show good contrast, with only the cuticle values on the boundary of the 1 in 1000 error margin.

Figure 5b shows that inter-tissue discriminability is poor, with PTC values falling well short of the “1 in 1000” guideline for automatic thresholding. These limitations and potential solutions will be considered in the discussion section.

Figure 5c–f shows the qualitative differences in overall scan quality between the various staining regimes. Sections from the different specimens were taken at approximately the same 2D plane across the thorax, however the orientation of the head is variable.

### Stain progression in all tissues

The stain progression for all tissue types and staining regimes is displayed across the five consecutive days in which they were scanned. This is with exception of the two iodine stained specimen, in which the contrast was insufficient to obtain reliable measurements.

**Figure 6:**
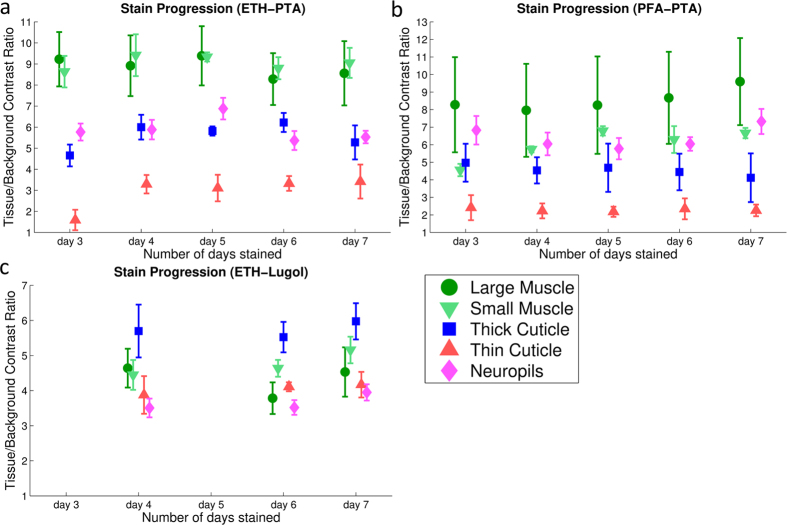
Contrast ratios between tissue and background for ETH-PTA (**a**), PFA-PTA (**b**), and ETH-Lugol (**c**) treatment, respectively. For each tissue type and treatment, the contrast ratios are plotted for staining days 3–7. The error bars shown represent one standard deviation of the contrast measurements taken (N = 8).

**Figure 7:**
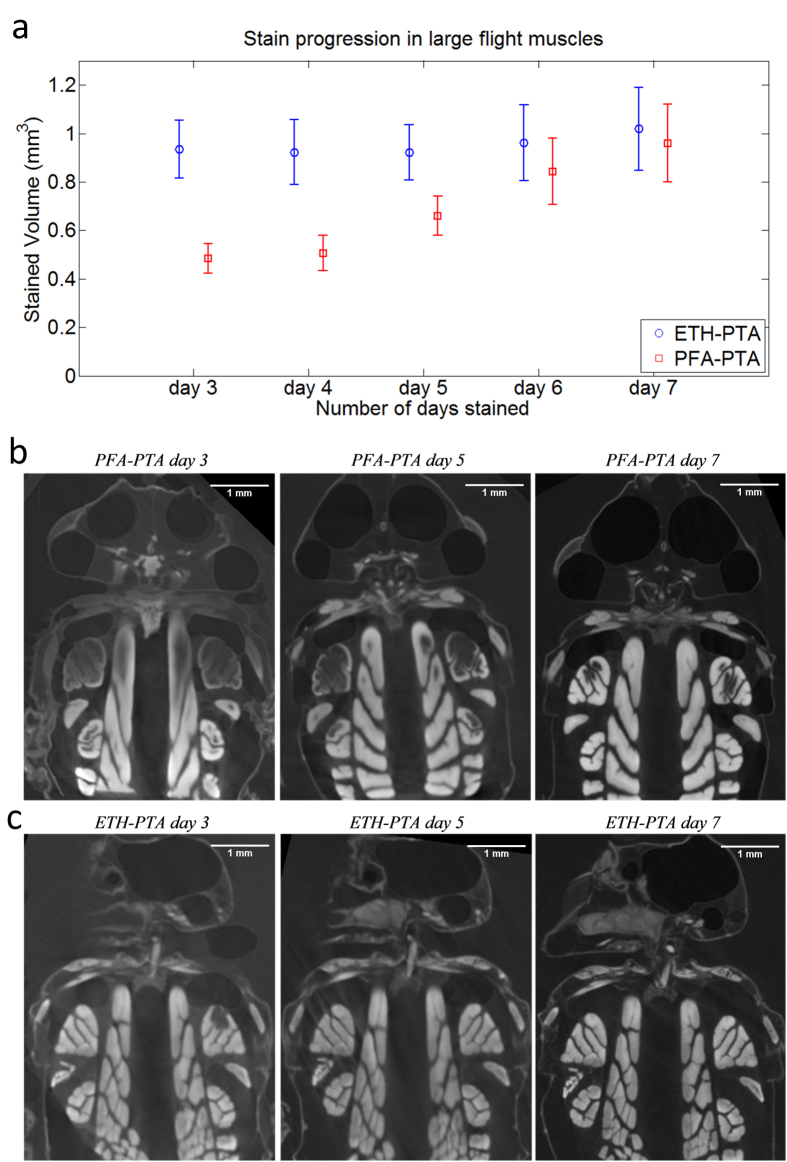
Effects of fixative on stain uptake. (**a**) Stained volume of the dorsoventral flight muscles against the number of days stained for ETH-PTA and PFA-PTA preparations. The error bars shown are calculated based on the uncertainty in the reconstructed voxel size; the focal spot size of the μCT scanner is 1 μm, therefore the error bars show the volume sizes for ±0.5 μm of the calculated volume. (**b**) Staining levels for the PFA-PTA specimen across days 3, 5 and 7. Each slice shown is from approximately the same 2D plane taken from the 3D volume. Each image is a 16-bit greyscale image; higher greyscale values indicate a higher level of staining. (**c**) Staining levels for the ETH-PTA specimen across days 3, 5 and 7.

The simple contrast ratio as defined in the methods section was used for all scanning conditions to determine stain progression as a function of the number of days stained. For the ETH-PTA specimen (Fig. 6a), the contrast ratio is approximately nine for the large and small muscles, 5.5 for the thick cuticle and neuropils and 3 for the thin cuticle. For the PFA-PTA specimen (Fig. 6b), the contrast ratio is approximately 9 for the large muscles, 6 for the small muscles, thick cuticle and neuropils and 2.5 for the thin cuticle. Whilst the values here are similar to ETH-PTA, the error bars for PFA-PTA stains of the large flight muscle are much larger. This is due to incomplete staining (see Fig. 7), and is further investigated below. For the ETH-Lugol specimen (Fig. 6c), the contrast ratio is approximately 6 for the thick cuticle, and 4 for the large muscles, small muscles, thin cuticle and neuropils. For the ethanol fixed cases (ETH-PTA, and ETH-Lugol), the greyvalue measurements remain constant throughout the 5 days of staining, suggesting that these tissues are fully stained by day 3 and do not overstain by day 7. The fly fixed in paraformaldehyde appeared to require longer to fully stain, as can be seen in Fig. 7, and is discussed below. The data for days 3 & 5 were lost due to significant movement artefacts during scanning which prevented reconstruction.

### Stain uptake

The stained volume of the large flight muscles was recorded in ethanol-fixed, and paraformaldehyde-fixed specimen, a weak vs. strong fixative respectively.

Figure 7a shows the level of staining in the right dorsoventral flight muscle for both PTA treatments to demonstrate the effect paraformaldehyde fixative has on the stain uptake rate within large tissues. The total stained volume for ETH-PTA was approximately 0.95 mm^3^ and remained constant throughout the 5 days of scanning. The stained vs. unstained volume ratio for PFA-PTA increased approximately linearly from 0.5 mm^3^/0.95 mm^3^ on day 3 up to the full 0.95 mm^3^ on day 7. Figure 7a and c show that the right dorsoventral fight muscle of the specimen fixed in ethanol is nearly 100% stained by day 3. The flight muscle of the paraformaldehyde-fixed specimen, however, is only approximately 50% stained after the same number of days (Fig. 6b and c). Direct comparison of PFA-PTA day 7 (Fig. 7b, panel 3) and ETH-PTA day 3 results (Fig. 7c, panel 1) shows a similar level of staining.

The stained volume of the right dorsoventral flight muscle was 0.95 mm^3^ in both PTA specimens. However, based on the 3 available measurements from the ETH-Lugol specimen, this muscle is much larger. Calculating the ratios suggests that the muscles stained with Lugol’s Solution were 37% larger (μ = 1.37, s.d. = 0.09, N = 3).

The uptake rate of the stains was measured at the eyes for ETH-PTA, PFA-PTA, and ETH-Lugol. For ETH-PTA: μ = 0.17 mm/day, N = 4, s.d. = 0.02), PFA-PTA: μ = 0.12 mm/day (N = 4, s.d. = 0.09). For ETH-Lugol, the entire fly was stained from day 3 onwards, suggesting a diffuse uptake rate of at least one order of magnitude higher than that of PTA preparation.

As there was no overstaining observed for any of the staining regimes, day 7 values were selected for the comparison presented in Fig. 5. Stain progression for the iodine-stained specimens could not be analysed, as the poor contrast resulted in unreliable measurements (see Fig. 5a).

## Discussion

All staining methods applied here gave enhanced contrast ratios compared to μCT studies in unstained tissue^2^,^17^. However, some stains clearly provided a better PTC than others, as demonstrated in Fig. 5a. Specifically, Lugol’s solution provided the best contrast between tissue and background, however different tissues tend to stain at similar levels, making inter-tissue discrimination more difficult. PTA provided better contrast between different tissue types, while still maintaining an acceptably high PTC between tissue and background. We can safely deem that the weak iodine solution is a comparatively inferior stain as the contrast values for both flies stained in low concentration (0.15%) iodine were several orders of magnitude lower than the other stains. Importantly, there was no overstaining for any of these preparations which would have lowered the contrast values with increasing time. Studies^3^,^6^ have shown that iodine-based stains tend to overstain, which could potentially have happened to our specimen, too, had we extended the staining period beyond 7 days.

We introduce a novel measure for quantifying the tissue-specific contrast based on a probabilistic approach. This method works equally effectively regardless of specimen preparation (i.e. different stains and fixatives) provided thorough staining of specimen. Our method provides an intuitive measure, the PTC value that is straightforward to interpret: A PTC value of 10^−3^ means that 1 in 1000 voxels will be misclassified when applying a simple threshold procedure to distinguish greyvalues of two different tissue types. If the PTC between two tissues relevant to a study is known beforehand, it will be clear whether subsequent segmentation can be done automatically or manually. A quantitative framework will allow for comparative studies to carried out using μCT, e.g. across different insect species. The disadvantage, however, is that a large sample size is required to calculate the PTC, since it is probabilistic in nature, stressing the need for effective and targeted staining regimes. The exact size required to use this method will depend on the dataset; certainly one cannot say with any authority that 1 in 1000 voxels have been incorrectly classified if the sample size is less than 1000, however the exact size required for will depend on the resolution of the CT scanner (and therefore the voxel size), the size of the tissues being measured, and the standard deviation of the greyvalues within those tissues.

Contrast is always defined relative to a reference value; which in this case would either be the background greyvalue or that of another tissue type. If the tissue type of interest is distant from other tissues and therefore contrasted only with the background greyvalues, then the tissue-background PTC is the most relevant (see Fig. 5a). This is quite often the case with larger structures such as the flight muscles. With the exception of the weak iodine stain, any of the other stains we have tested provide sufficiently high contrast to easily segment out any of the selected tissue types from the background. If the tissue type of interest is surrounded by other tissues that need to be discriminated against e.g. the neck motor system where muscle and cuticle are in close proximity, then the PTC between tissues is most relevant (see Fig. 5b). In this case, all of the inter-tissue PTC values fall well short of the 10^−3^ benchmark required for automatic thresholding, regardless of staining regime. The consequence is that segmenting out neighbouring structures would require additional user input.

We found that the type of fixative used, either alcohol or paraformaldehyde (PFA), has little effect quantitatively on the resulting contrast at the end of the staining period, however it appears to have a substantial impact on the speed at which the contrast agent is taken up. The use of PFA as fixative slowed down the stain uptake by approximately 50% compared to fixation with ethanol. Furthermore, the stain uptake rate measured at the eyes showed that tissue fixed with PFA will uptake PTA approximately 70% as quickly as the same tissue fixed in ethanol. Although we did not obtain precise measures of stain uptake rates, it is safe to conclude that PFA as a fixative slows down the stain uptake rate by approximately a factor of two. While both of these fixatives causes shrinkage, ethanol has the distinct advantage of being ideally suited to scanning museum specimen, as well as its faster stain uptake rate. To customise an optimal staining timeline, a balance has to be struck between making the incision sufficiently far away from the scanning region of interest so as not to damage any relevant structures whilst ensuring it is close enough to these structures to minimise staining time and ensure thorough tissue penetration.

When estimating *in vivo* volumes of tissue from these measurements, it is important to take into consideration that ethanol and paraformaldehyde dehydration of tissue causes shrinkage^3^. Furthermore, a comparison of the results obtained from Lugol’s and PTA-stained specimen (Fig. 5) reveals that the Lugol-stained muscles swell to 137% of the size of the PTA-stained specimen’s muscles. One possible explanation for the observed volume difference is the potential for swelling due to water-based Lugol’s solution as opposed to shrinkage caused by ethanol-based staining solutions. It therefore seems likely that the ethanol-based and the Lugol’s-based measurements provide the lower and upper bounds of the large flight muscle *in vivo* volumes, respectively.

With appropriate staining as outlined in this study, and due to the semi-automatic nature of the segmentation procedure, this method can achieve extremely high data throughput. As we have successfully applied the methodology reported here in preliminary experiments to other Dipteran flies and Lepidoptera (*Manduca*), we are confident that it will be suitable for application to a variety of insects and other arthropods.

## Additional Information

**How to cite this article**: Swart, P. *et al*. A quantitative comparison of micro-CT preparations in Dipteran flies. *Sci. Rep.* **6**, 39380; doi: 10.1038/srep39380 (2016).

**Publisher's note:** Springer Nature remains neutral with regard to jurisdictional claims in published maps and institutional affiliations.

## Figures and Tables

**Table 1:** Table of fixatives and stains.

Abbreviation	Fix	Stain	Notes
ETH – PTA	Ethanol	PTA	Fixed in 100% ethanol, stained in 0.5% PTA solution.
PFA - PTA	PFA	PTA	Fixed in 2% PFA solution, stained in 0.5% PTA solution.
ETH - Iodine	Ethanol	Iodine	Fixed in 100% ethanol, stained in 0.15% iodine solution.
PFA - Iodine	PFA	Iodine	Fixed in 2% PFA solution, stained in 0.15% iodine solution.
ETH - Lugol	Ethanol	Lugol’s Iodine	Fixed in 100% ethanol, stained in 1% Lugol’s Iodine.
